# Studying the Specific Activity of the Amide Form of HLDF-6 Peptide using the Transgenic Model of Alzheimer’s Disease

**Published:** 2017

**Authors:** A. P. Bogachouk, Z. I. Storozheva, G. B. Telegin, A. S. Chernov, A. T. Proshin, V. V. Sherstnev, Yu. A. Zolotarev, V. M. Lipkin

**Affiliations:** Shemyakin–Ovchinnikov Institute of Bioorganic Chemistry, Russian Academy of Sciences, Miklukho-Maklaya Str., 16/10, Moscow, 117997, Russia; V. Serbsky Federal Medical Research Center of Psychiatry and Narcology, Ministry of Health, Kropotkinskiy Lane, 23, Moscow, 119034, Russia; Branch of the Shemyakin–Ovchinnikov Institute of Bioorganic Chemistry, Russian Academy of Sciences, Nauki Ave., 6, Moscow oblast, 142290, Russia; Anokhin Institute of Normal Physiology, Russian Academy of Sciences, Baltiyskaya Str., 8, Moscow, 125315, Russia; Institute of Molecular Genetics, Russian Academy of Sciences, Kurchatov Sq., 2, Moscow, 123182 , Russia

**Keywords:** Differentiation factor HLDF, amide form of HLDF-6 peptide, neuroprotective and nootropic activities, Alzheimer’s disease, transgenic mice

## Abstract

The neuroprotective and nootropic activities of the amide form (AF) of the HLDF-6 peptide (TGENHR-NH_2_) were studied in transgenic mice of the B6C3-Tg(APPswe,PSEN1de9)85Dbo (Tg+) line (the animal model of familial Alzheimer’s disease (AD)). The study was performed in 4 mouse groups: group 1 (study group): Tg+ mice intranasally injected with the peptide at a dose of 250 μg/kg; group 2 (active control): Tg+ mice intranasally injected with normal saline; group 3 (control 1): Tg- mice; and group 4 (control 2): C57Bl/6 mice. The cognitive functions were evaluated using three tests: the novel object recognition test, the conditioned passive avoidance task, and the Morris water maze. The results testify to the fact that the pharmaceutical substance (PhS) based on the AF of HLDF-6 peptide at a dose of 250 μg/kg administered intranasally efficiently restores the disturbed cognitive functions in transgenic mice. These results are fully consistent with the data obtained in animal models of Alzheimer’s disease induced by the injection of the beta-amyloid (βA) fragment 25-35 into the giant-cell nucleus basalis of Meynert or by co-injection of the βA fragment 25-35 and ibotenic acid into the hippocampus, and the model of ischemia stroke (chronic bilateral occlusion of carotids, 2VO). According to the overall results, PhS based on AF HLDF-6 was chosen as an object for further investigation; the dose of 250 μg/kg was used as an effective therapeutic dose. Intranasal administration was the route for delivery.

## INTRODUCTION

Cerebrovascular and neurodegenerative diseases, the major cause of mortality and disability in Russia and worldwide, are among the current medical social problems ranking high on the agenda. The most common disorder, Alzheimer’s disease (AD), is a neurodegenerative disorder diagnosed in almost 44 million people [1]. AD progresses slowly but inevitably results in dysfunction of the key organ, the brain, and a number of other systems of the human body. Alzheimer’s disease has been recognized as one of the major four medical social issues of contemporary society.

Ischemic stroke (IS) is one of the most severe cerebrovascular diseases. More than 15 million stroke cases are reported annually [2], including over 450,000 cases in Russia. Adverse side effects, tolerance, and lack of effectiveness are the significant drawbacks of the drugs used to manage AD and IS that substantially narrow their application. All these factors call for urgent measures: elaborating and launching into clinical practice novel effective drugs for the prevention and treatment of these diseases.

In 1994, we discovered the human leukemia differentiation factor (HLDF) and isolated it from a culture medium of HL-60 cells treated with retinoic acid [3]. The six-membered fragment TGENHR (HLDF-6 peptide), which totally reproduces the differentiation activity of the full-length factor and exhibits a broad range of nootropic and neuroprotective activities, was identified when studying HLDF. Direct evidence to the neuroprotective effect of HLDF-6 peptide was obtained for a primary culture of hippocampal and cerebellar neuronal cells, as well as immunocompetent cells. This peptide exhibits an anti-apoptotic activity and protects cells against beta-amyloid (βA) peptide, sodium azide, ceramide, ethanol, cold stress, and hypoxia. HLDF-6 peptide enhances the viability of early mouse embryos *in vitro* [4-7].

An evaluation of the effect of HLDF-6 peptide using various experimental animal models (the Morris water maze, the passive avoidance, delayed matching to position, and the recognition memory tests) demonstrated that central and systemic administration of the peptide to healthy animals enhances the formation and storage of long-term memory. The peptide was shown to eliminate the pronounced cognitive deficit in experimental models of clinical pathology (AD and IS) and to contribute to the restoration of the disturbed memory [8, 9]. The administration of HLDF-6 to animals with chronic cerebral ischemia ensures a reliable neuroprotective effect as it protects cerebral neurons against death in ischemic conditions [10].

Investigation of the pharmacokinetics of HLDF-6 peptide has demonstrated that the peptide is extremely unstable in an animal organism: its half-life in rat plasma is 2 min. HLDF-6 is hydrolyzed starting at its C-end; dicarboxypeptidases make a major contribution to it [11]. Amidation of the C-terminal carboxylic group was used to protect the peptide against dicarboxypeptidases. The half-life of the amide form (AF) of HLDF-6 peptide (TGENHR-NH_2_) in rat plasma was shown to be 8 min, significantly higher than that of the native form (NF) of the peptide (TGENHR-OH) [12].

In order to choose the most effective form of HLDF- 6 peptide for its investigation as a pharmacological substance (PhS), we conducted an extended comparative study of the neuroprotective and nootropic activities of FS samples based on the AF and NF of HLDF-6 peptide in animal models of AD and IS. At the first stage, we revealed the neuroprotective and nootropic activities of the PhS based on HLDF-6 peptide in models of sporadic Alzheimer’s disease. The models used were as follows: a) cognitive deficit induced by injection of beta-amyloid 25–35 fragment to the giant-cell nucleus basalis of Wistar rats; b) cognitive deficit induced by co-injection of beta-amyloid 25–35 fragment and ibotenic acid to the hippocampus of Wistar rats. A comparative analysis of the data obtained using both AD models demonstrated that the neuroprotective effect of the AF of HLDF-6 peptide evaluated from the degree of restoration of the disturbed cognitive function was significantly higher than that of the NF of peptide. An almost complete function restoration was observed when using the AF of HLDF-6 peptide at a dose of 250 μg/kg (a much lower dose than those of comparator agents) [12].

We report on the results of a study of the specific activity of PhS based on the AF of HLDF-6 peptide using a transgenic model of AD. The transgenic model was used in accordance with the Guidelines for Preclinical Studies of Nootropic Drugs [13].

Alzheimer’s disease is a neurodegenerative disorder characterized by cognitive impairment and dementia. The familial and sporadic forms of AD are differentiated. Familial AD has an autosomal dominant inheritance pattern. In 1991, the first gene causing familial AD was identified: the mutant gene of the amyloid precursor protein (APP) residing in chromosome 21 [14]. Mutations in other genes that increase the risk of AD were detected later. Among the products of these genes, the strongest effect was observed for presenilin-1, which is responsible for 70–80% of early-onset familial AD cases, with its gene residing in chromosome 14 [15]. The creation of transgenic animals allows one to simulate the molecular processes of AD development during the entire life of an organism. The key advantage of the transgenic model is that insertion of human genes coding for the development of familial AD (the *APP *and presenilin genes) to animals results in the development of pathogenetic processes in the animals that are similar to manifestations of AD in humans (amyloid plaque formation, oxidative stress, disruption of cholinergic transmission, and neuronal death). This provides grounds for suggesting that the processes taking place in the central nervous system of the model animals are similar to those occurring during the development of AD in humans. The so-called B6C3- Tg(APPswe,PSEN1de91)85Dbo double transgenic mice are the best choice for studying potential drugs [16]. Animals of this line express the mutant human presenilin and chimeric mouse/human amyloid protein. A typical feature of this line is early (at the age of 6 or 7 months) development of an Alzheimer-like pathology caused by accelerated βA deposition and cognitive impairment in the brain, which is evaluated using spatial learning tests [17, 18].

Our study aimed to evaluate the neuroprotective and nootropic activities of the AF of HLDF-6 peptide in B6C3–Tg(APPswe,PSEN1de91)85Dbo transgenic mice, an animal model of familial AD.

## EXPERIMENTAL

**Synthesis of the AF of HLDF-6 peptide**

The AF of the peptide was synthesized according to the procedure described in [12].

**Experimental animals**

Healthy male B6C3-Tg(APPswe,PSEN1de9)85Dbo (Tg+) mice, wild-type B6C3 (Tg-) mice, and C57Bl/6 mice were used. Eight-month-old mice weighing 28–35 g were obtained from the laboratory animal breeding nursery of the Pushchino Branch of the Institute of Bioorganic Chemistry (Russian Academy of Sciences) that has earned international AAALACi accreditation. The quality control system for the production of laboratory animals has been certified to comply with the international standard requirements ISO 9001:2008. All the experiments using animals were conducted in accordance with the Guidelines for Good Laboratory Practice of the Russian Federation (Order no. 708n of the Ministry of Healthcare and Social Development of the Russian Federation dated August 23, 2010, Moscow, “On Approval of the Guidelines for Good Laboratory Practice”) and with the recommendations provided in the Guidelines for Preclinical Studies of Nootropic Drugs [13]. The mice were divided into four groups, with 10 mice per group: group 1 (experimental group) included Tg+ mice that intranasally received the PhS at a dose of 250 μg/g; group 2 (active control) consisted of Tg+ mice that intranasally received normal saline; group 3 (control 1) consisted of Tg- mice that intranasally received normal saline; and group 4 (control 2) included C57Bl/6 mice that intranasally received normal saline. The additional control group was used because several models of cognitive function were included in the experiment. An analysis of published data demonstrates that the learning and memory features in B6C3-Tg(APPswe,PSEN1de9)85Dbo mice have been evaluated mostly using spatial learning tests, while the other cognitive models have been studied insufficiently [18, 19]. The findings obtained using the spatial learning tests demonstrate that the differences between B6C3-Tg+ and B6C3-Tg- mice are most pronounced in models exposed to a high stress level (e.g., in the Morris water maze rather than in the Barnes maze test) [20]. Meanwhile, the differences between B6C3-Tg+ and B6C3-Tg- mice were detected mostly in models with positive rather than negative reinforcement [21]. The cognitive abilities of B6C3-Tg- also have not been fully characterized. In this context, we deemed it reasonable to use the group of additional control to evaluate the validity of the experimental protocols. This group consisted of C57Bl mice that had an appreciably high level of orientational and exploratory activity and stress resistance [22] and near-average cognitive abilities [23].

No comparator drug was used, since the action of clinically effective agents (memantine, donepezil, etc.) for this model is still being tested in pilot studies and has not been characterized sufficiently well [24, 25].

**Protocols of PhS administration and testing of the cognitive functions**

Group 1 animals intranasally received the AF of the peptide at a dose of 250 μg/kg (10 μL/kg) in each nostril every other day for 30 days (a total of 15 injections). Group 2–4 animals received normal saline according to the same scheme. The cognitive function was assessed after the injections had been completed using the following scheme: days 3–5, the novel objection recognition test; days 8–10, the passive avoidance test; and days 13–17, the Morris water maze test.

**Novel object recognition test**

The novel object recognition test was conducted in a 35 × 35 × 40 cm chamber made of gray plastic under room light. The test consisted of three five-minute sessions separated by a 24 h interval: 1 – without objects to allow a mouse to adapt to the apparatus; 2 – with two equal objects: metal cylinders 3 cm in diameter and 3 cm high; 3 – one of the cylinders was replaced with a plastic cube (3 cm edge length). Animal behavior was recorded using a digital video camera and analyzed using the EthoVision XT software (Noldus). The levels of orientational and exploratory activity were assessed when the mice were exploring the objects for the first time (session 2) and when exploring the “familiar” and the “novel” objects in session 3. The recognition index was calculated using the formula (*T*_n_ – *T*_f_ / *T*_n_ + *T*_f_) × 100%, where *T*_n_ is the exploration time of the novel object, and *T*_f_ is the exploration time for the familiar object during session 3 [26-28].

**Passive avoidance test**

The passive avoidance test was conducted in an apparatus manufactured by Columbus Instruments (USA). The experimental chamber consisted of two identical compartments 25 × 40 × 25 cm in size with a grid-metal floor. The compartments were connected through a hole in the common wall (8 × 8 cm) equipped with guillotine doors. One of the compartments was lit, while the other one was dark. During passive avoidance training, an animal was placed into the lit compartment and the latency prior to it entering the dark compartment (emergence of the hole reflex) was recorded. Immediately after all four paws of the animal were in the dark side of the chamber, the compartments were separated by the guillotine doors. The mouse was subjected to electrocutaneous irritation through the floor grid (0.6 mA, 3 s), then it was immediately taken out of the chamber and placed into its home cage. The acquired response was tested 48 h after it had been established. The mouse was placed into the lit compartment again, and the latency prior to it entering the dark side was measured [29-31].

**Morris water maze test**

The Morris water maze was a circular gray pool 165 cm in diameter, with walls 60 cm high, filled with water to a level of 40 cm. A round plexiglass platform 9 cm in diameter was submerged 2 cm below the water level in the center of one of the sectors. The pool was placed in a stimulus-rich environment (posters, cabinets, etc.), without any key stimuli located above the platform. During the training session, the animals were placed in water at four different locations and the time taken to reach the platform was recorded. Once the animal had reached the platform, it was left there for 15 s and then returned back into its home cage for 2 min. Training was performed during 5 days [18].

**Statistical analysis**

Statistical analysis of the results was carried out using the nonparametric Mann–Whitney test. The STATISTICA 6.0 software was used for the analysis.

**Table:** Parameters of orientation and exploratory activity of mice in different groups in the novel object recognition test

Animal group	Total object exploration time during the testing phase (test day 2), s			Z values (standardized Mann– Whitney U-test) and significance of intergroup differences
	Lower quartile	Median	Upper quartile	
1. Tg+ with PhS injected	10.2	13.4	17.4	#Z = 0.22, p = 0.83 *Z = 1.55, p = 0.12
2. Tg+ with normal saline injected	7.7	12.3	14.1	*Z = 2.41, p = 0.0156
3. Tg- with normal saline injected	15.4	16.0	17.3	&Z = 1.06, p = 0.29
4. C57Bl with normal saline injected	10.1	16.6	20.2	

^#^ – Statistical significance of the difference from group 2.

^*^ – Statistical significance of the difference from group 3.

^&^ – Statistical significance of the difference from group 4.

## RESULTS AND DISCUSSION

**Novel object recognition test**

**Fig. 1:**
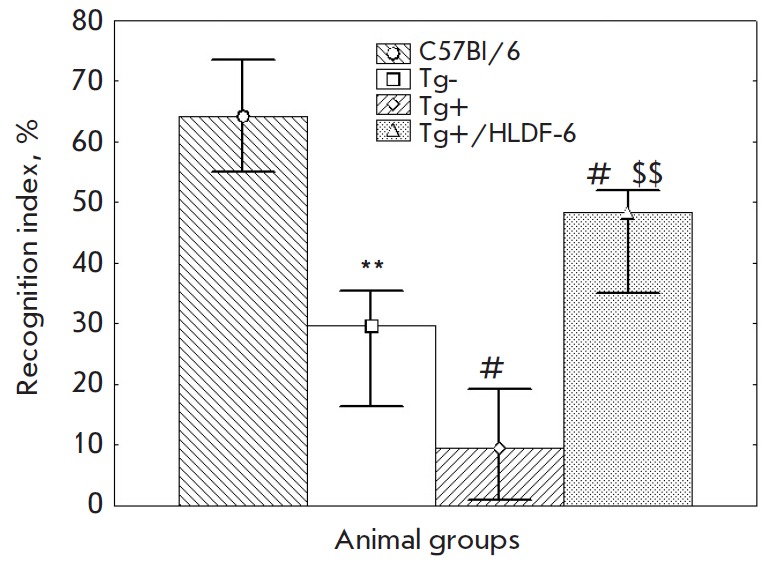
Indices of long-term memory in the model of object recognition test in mice of different groups. The data are presented as the median, the upper, and lower quartiles. * – p < 0.05, ** – p < 0.01 compared to the C57Bl/6 group; # – *p * < 0.05 compared to the Tg- group; and $$ – p < 0.01 compared to the Tg+ group.

Exploration of objects during the testing session (*Table*) showed no differences in orientational and exploratory activity between the Tg- and C57Bl/6 control groups. Meanwhile, the animals in the active control group (Tg+ with normal saline injection) showed a significantly lower exploratory activity than that in the Tg-group. The study group animals differed significantly from neither the active control group nor Tg- mice. Appreciably high object recognition indices characterizing explicit long-term memory related to the function of the parahippocampal cortex (the region of the middle temporal gyrus) in C57Bl/6 mice were revealed; these indices were comparable to the published data [32]. The recognition index was significantly decreased in animals of the control group Tg- vs the C57Bl/6 group and in active control group mice vs. the Tg- control group mice. Injection of the peptide-based PhS restored the recognition index to a level higher than the values both in the active control and the Tg- groups (*Fig. 1*).

**Passive avoidance model**

No statistically significant intergroup difference in latency prior to entering the dark compartment was detected on training day before the mice were subjected to electrocutaneous irritation.

**Fig. 2:**
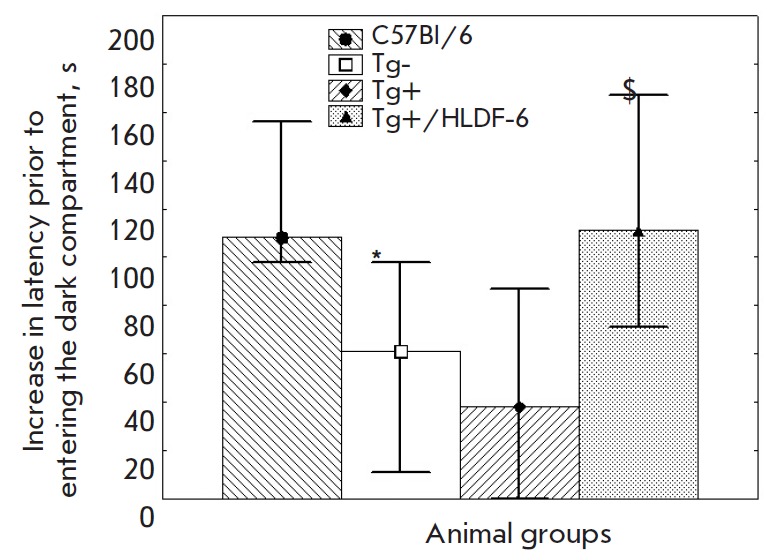
Indices of learning in the passive avoidance test in mice of different groups. Y-axis – the increase in latency prior to entering the dark compartment at the training session compared to the testing one (s). The data are presented as the median, the upper, and lower quartiles. * – p < 0.05 compared to the C57Bl/6 group and $ – p < 0.05 compared to the Tg+ group.

Meanwhile, a significant intergroup difference in the increase in latency prior to entering the dark compartment was revealed on testing day, which characterized long-term memory (*Fig. 2*).

A statistically significant difference in the increase in latency prior to entering the dark compartment on testing day characterizing long-term memory was revealed neither in the control C57Bl/6 and Tg- groups nor between the active control and the Tg- group. The animals that had received PhS were significantly superior to the active control group and showed a tendency (*p *= 0.062) to be superior to the Tg- group.

**Spatial memory in the Morris water maze model**

**Fig. 3:**
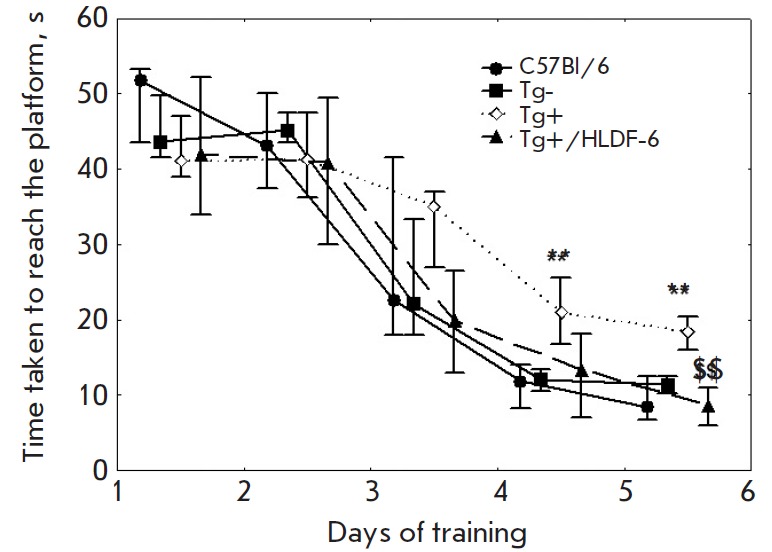
The dynamics of Morris water maze training of mice. The data are presented as the median, the upper, and lower quartiles. ** – p < 0.01 compared to the Tg-group; $$ – p < 0.01 compared to the Tg+ group.

The average latency to reach the platform on training days 2–5 was an index of long-term memory in this model. The results are shown in *Fig. 3*.

A statistically significant difference between the control (C57Bl/6 and Tg-) groups was revealed by the Mann–Whitney test in none of the training days. The Tg+ animals that had been injected with normal saline showed a significant spatial memory deficit on training days 4 and 5 compared to the Tg- group. Administration of PhS partially restored spatial memory in Tg+ mice. On training day 4, the maze performance was intermediate with respect to that in Tg+ animals that had received normal saline and Tg- animals. On training day 5, the Tg+ mice that had been injected with PhS performed the task much better than the Tg+ group injected with normal saline, while showing no significant difference with respect to the control group Tg-.

## CONCLUSIONS

Hence, the results demonstrate that PhS based on the AF of HLDF-6 at a dose of 250 μg/kg delivered intranasally effectively stimulated the performance of cognitive tasks by transgenic B6C3-Tg(APPswe,PSEN1de9)85Dbo mice in all the tests used. It is noteworthy that the results obtained in the additional control group of C57Bl/6 mice verify the validity of the models of cognitive functions used in our study. In the model of spatial acquisition in an enriched environment (the Morris maze), the dynamics of training of the control Tg- group did not differ from that among C57Bl/6 mice. In the active control group, the dynamics of spatial acquisition was reduced compared to that in the Tg- group, while administration of PhS had a pronounced neuroprotective effect and restored the indices of spatial acquisition to their control level.

In the novel object recognition test, the learning parameters in the Tg- group significantly decreased compared to those in the C57Bl/6 group; a less pronounced reduction was also observed in the active control group with respect to the Tg- group. Administration of PhS increased the learning index to a level exceeding that in the Tg- group.

A decrease in the learning indices in the Tg- group with respect to the C57Bl/6 group was also observed in the passive avoidance test. However, unlike in other tests, the learning ability in animals in the active control group was not worse compared to that in the Tg- group. Like in other models, administration of PhS stimulated long-term memory in transgenic mice up to a level that was even somewhat higher than that in the control groups.

A combination of the results indicates that PhS based on the AF of HLDF-6 peptide has both neuroprotective and nootropic properties; i.e., it stimulates the cognitive function regardless of whether there is a neurodegenerative process or not.

The results of our study are fully consistent with the data obtained for animal models of AD: the βA fragment (25–35) was injected into the giant-cell nucleus basalis of Meynert or the βA fragment and ibotenic acid were co-injected into the hippocampus [12]. According to the overall results, PhS based on the AF of HLDF-6 was chosen as an object for further clinical studies; the dose of 250 μg/kg was used as an effective therapeutic dose. Intranasal administration was the route for delivery.

We had previously studied the contribution of serotonin, GABA, and NMDA glutamate brain receptors to the nootropic effect of the AF form of HLDF- 6 peptide by radioreceptor assay. These receptors are involved in the pathogenesis of various neurological disorders and chronic neurodegenerative diseases [33, 34]. The effect of the AF of HLDF-6 peptide on the parameters of binding between radiolabeled ligands and NMDA receptors on hippocampal membranes and between GABA-A and 5HT2A serotonin receptors on the membranes of the prefrontal cortex in BALB/c mice was investigated. Subchronic injection of the peptide into the murine hippocampus was shown to increase the amount of the ligand (G-3H MK-801) that bound [11] only for the NMDA glutamate receptors, an indication of the density of the corresponding receptors. Hence, the AF of HLDF-6 peptide restores the amount of NMDA receptors to its normal level, thus improving cognitive behavior. Subchronic fivefold injection of the AF of HLDF-6 peptide had no effect on the densities of GABA receptors and nicotinic cholinoreceptors but was accompanied by a decrease in the density of 5-HT2 serotonin receptors [35]. A conclusion was drawn that the mechanism of formation of the neuroprotective activity of Thr-Gly-Glu-Asn-His-Arg-NH2 peptide may involve an effect on the glutamate and serotoninergic systems.

HLDF-6 peptide is a fragment of the natural differentiation factor HLDF-6 that is present in blood and the central nervous system of mammals and humans. The preclinical studies of the pharmaceutical substance based on the AF of HLDF-6 peptide have demonstrated that it is satisfactorily soluble, easily metabolized, non-immunogenic and nontoxic, characterized by a high effectiveness of specific activity, and safe at a dose tenfold higher than the therapeutic dose. The results of preclinical studies provide grounds to hope that the pharmaceutical substance will successfully pass clinical trials. In this case, one can anticipate that the agent will become widely used in the therapy of AD.

## Abbreviations

HLDF: human leukemia differentiation factor
AF: amide form
NF: native form
AD: Alzheimer’s disease
IS: ischemic stroke
PhS: pharmaceutical substance
βA: beta-amyloid
